# Wisdom Once Gained Is Not Easily Lost: Implicit Theories About Wisdom and Age-Related Cognitive Declines

**DOI:** 10.1093/geroni/igaa010

**Published:** 2020-05-04

**Authors:** Sarah J Barber, Dina Kireeva, Jordan Seliger, Eranda Jayawickreme

**Affiliations:** 1 Department of Psychology, San Francisco State University, California; 2 Department of Psychology, Georgia State University, Atlanta; 3 Department of Psychology, Wake Forest University, Winston-Salem, North Carolina

**Keywords:** Alzheimer’s disease, Cognitive aging, Folk beliefs, Lay definitions, Wise person

## Abstract

**Background and Objectives:**

Most people agree that cognitive capabilities are an integral component of wisdom and its development. However, a question that has received less attention is whether people view maintaining cognitive capabilities as a necessary prerequisite for maintaining wisdom.

**Research Design and Methods:**

This study used a mixed-methods approach to evaluate people’s views about the relationship between age-related cognitive declines, Alzheimer’s disease (AD), and wisdom. Our final sample of 1,519 adults ranged in age from 18 to 86.

**Results:**

The majority of participants stated that wisdom could be present even in people with significant age-related cognitive declines or with AD. In the qualitative responses, common justifications for this were (a) that even people with severe AD can still exhibit wise behaviors during lucid moments, (b) that wisdom is an immutable characteristic that is impossible to lose, and (c) that wisdom maintenance and cognitive capability maintenance are separate constructs.

**Discussion and Implications:**

Although prior research has examined implicit theories about the role of cognition in the development of wisdom, this is the first study to examine implicit theories about whether cognitive declines lead to wisdom declines. The results suggest that most people hold essentialist beliefs about wisdom, viewing it as a fixed and unchangeable trait rather than as a malleable skill.

**Translational Significance**

Most people believe that cognitive declines and dementia do not necessarily lead to losses of core qualities, including wisdom. This continued sense of self may be a beneficial coping strategy that can assist in enhancing well-being and self-esteem.

How is wisdom developed and maintained over the life span? To answer this, researchers often examine peoples’ commonly held beliefs about the core qualities of wisdom and how they develop (i.e., assess implicit theories of wisdom; for reviews, see Bluck & Glück, 2005; Phan, Blackie, Horstmann, & Jayawickreme, in press; Staudinger & Glück, 2011). This can be done by asking people to generate or evaluate the characteristics associated with wisdom (e.g., Clayton & Birren, 1980; Glück & Bluck, 2011; Holliday & Chandler, 1986; Sternberg, 1985; Takahashi & Bordia, 2000), by asking them to nominate and describe a person they believe to be wise (e.g., Denny, Dew, & Kroupa, 1995; Glück, Bischof, & Siebenhüner, 2012; Weststrate, Ferrari, & Ardelt, 2016), by asking them to describe a time that they themselves were wise (e.g., Bluck & Glück, 2004; Glück, Bluck, Baron, & McAdams, 2005), or by asking them to judge the wisdom of fictional characters who differ in abilities, age, or gender (e.g., Hira & Faulkender, 1997; Knight & Parr, 1999; Sternberg, 1985). These approaches give insight into “people’s basic assumptions about themselves and their world” (Dweck, 1996, p. 69), and as such, give insight into the beliefs that guide people’s lives.

Even though there is variability in how wisdom is defined by people from different age groups (e.g., Clayton & Birren, 1980; Glück & Bluck, 2011), professions (Sternberg, 1985), and cultures (e.g., Takahashi & Bordia, 2000; for a review, see Grossmann & Kung, in press), across studies there has been seemingly unanimous agreement that cognitive capabilities are a central component of wisdom. People typically see wise people as having a depth of experiences and an extraordinary scope of life knowledge (i.e., as possessing high levels of crystallized cognitive capabilities), and as having superior abilities in thinking logically to solve new problems faced by themselves and others (i.e., as possessing high levels of fluid cognitive capabilities; Bluck & Glück, 2005). The notion that cognitive capabilities are a key aspect of wisdom is present even in elementary-school-aged children (e.g., Glück et al., 2012), and also has parallels to how researchers have defined wisdom in their explicit theories (Jeste et al., 2010). For instance, Ardelt (2000, 2003) proposed that cognition is one of the three key aspects of wisdom. Moreover, in the Berlin wisdom paradigm, wisdom is defined as an intellectual virtue. It is viewed as expert knowledge in the interpretation and management of important aspects of life (i.e., in the “fundamental pragmatics of life”; Baltes & Smith, 1990), and is assumed to rely upon both crystallized knowledge and fluid cognitive capabilities (Baltes & Smith, 2008; Baltes & Staudinger, 2000). More recently, research on wise reasoning has emphasized other cognitive capacities such as owning one’s limitations and exhibiting intellectual humility (e.g., Brienza et al., 2018; Grossmann, 2017; Zachry, Phan, Blackie, & Jayawickreme, 2018).

Although cognitive capabilities are viewed as a necessary component of wisdom, both implicit and explicit theories tend to agree that cognitive capabilities—in and of themselves—are not sufficient for the development of wisdom (Baltes & Staudinger, 2000; Grossmann, 2017; Jeste et al., 2010; Staudinger & Glück, 2011). However, a question that has received less attention is whether maintaining cognitive capabilities is a necessary prerequisite for maintaining wisdom. Although research has shown that the extent to which people make wise judgments in a laboratory task is only weakly related to measures of cognitive capabilities (Staudinger, Lopez, & Baltes, 1997), wise judgments may require that people maintain at least a certain level of cognitive capabilities. For example, Baltes, Staudinger, Maercker, and Smith (1995) found that wisdom-related performance was lower in older-old adults, who presumably had experienced greater age-related cognitive declines, than in younger-old adults. This is consistent with some explicit theories that suggest that age-related declines in cognitive capabilities relate to declines in wisdom (for reviews, see Staudinger, 1999; Sternberg, 2005). However, to the best of our knowledge, no prior research has assessed implicit theories about this relationship. The goal of the current study was to examine this issue. Do people believe that you can be wise if you have experienced age-related cognitive declines and are prone to “senior moments?” Do people believe that you can be wise if you have a diagnosis of Alzheimer’s disease?

On the one hand, given that cognitive capabilities are often viewed as an integral component of wisdom, it is possible that people will see age-related cognitive declines (Craik & Salthouse, 2011) as leading to wisdom declines. This would be consistent with research showing that even though “wise,” “incompetent,” and “senile” are all stereotypes associated with older adults, these characteristics are not necessarily used to describe the same person. Whereas older adults associated with the substereotype of “Perfect Grandparent” are considered wise, those associated with the substereotype of “Severely Impaired” are considered incompetent and senile (Hummert, Garstka, Shaner, & Strahm, 1994).

On the other hand, recent research has suggested that people (at least in North America) often hold essentialist beliefs about wisdom and view it as a fixed and unchangeable trait rather than as a malleable skill (Grossmann, in press). For example, people tend to agree that wisdom is innate in a person, is based upon their biological predisposition, and that the amount of wisdom people possess cannot be changed (Grossmann, Gerlach, & Denissen, 2016, as cited in Grossmann & Kung, in press). Similar views are sometimes even espoused by wisdom researchers. In a survey of wisdom researchers, there was, on average, slight agreement with the notion that wisdom is a trait that, to some degree, is present in everyone (Jeste et al., 2010). Likewise, some (but not all) wisdom researchers invoke essentialist characteristics when describing wisdom (Grossmann, 2017). Based upon these findings, it is possible that people will believe that wisdom can be present in anyone, and cannot be erased via age-related cognitive declines or Alzheimer’s disease.

## Current Study

This is the first study to systematically examine implicit theories about the relationship between wisdom, cognitive decline, and dementia. To do so, we used a mixed-methods approach. Participants rated the likelihood that people can possess wisdom given age-related declines in cognitive capabilities or a diagnosis of Alzheimer’s disease (AD) and then had the opportunity to justify their responses.

## Research Design and Methods

### Participants

Participants were residents of the United States who were recruited using the panel service from www.TurkPrime.com (Litman, Robinson, & Abberbock, 2017). Participants recruited through this service have had minimal prior exposure to psychological studies. In total, 1,965 individuals consented to participate. However, from this sample, we eliminated 446 responses: 199 who consented but did not complete the survey, 10 whose responses were associated with duplicate IP addresses, 47 who provided incompatible birth years and ages, 179 who provided an incorrect response to an attention check (see Method section), and 11 who failed to answer one or more of the questions assessing implicit wisdom theories (see Measures section). This left a final sample size of 1,519 participants.

Within this final sample, participants ranged in age from 18 to 86 (*M* = 47.76, *SD* = 15.46) with 71.1% self-identified as female, 26.9% as male, and 0.3% as genderqueer or nonbinary. Participants self-identified the following ethnic/racial identities: 78.2% white, 5.2% black, 4.1% Asian, 4.0% Hispanic/Latinx, 3.8% as Biracial or Multiracial, 0.8% Native American or Alaskan Native, 1.7% as another race or ethnicity, and 2.2% who declined to state. They also had a broad range of educational backgrounds: 1.4% had less than a high school degree, 19.5% had a high school diploma or GED, 21.7% had some college, 13.1% had a 2-year college degree, 26.8% had a 4-year college degree, 12.2% had a Master’s degree, 1.1% had a PhD, and 2.4% had a JD or MD degree.

### Measures

Participants first provided a definition of wisdom in response to the following question adapted from Glück and Bluck (2011): There are many human virtues—honesty, courage, intelligence, and others. Wisdom is a unique quality that differs from all others. What do you consider to be the essential aspects of wisdom? We included this open-ended question to ensure that participants first spent time thinking about how they personally defined wisdom and were oriented to think of it as different from other character traits.

Participants next answered the following three questions on a 1 (*definitely yes*) to 5 (*definitely no*) scale. These served as our quantitative outcomes.

1. In your opinion, can someone who has experienced cognitive decline and who is prone to “senior moments” be wise?
2. In your opinion, can someone who has been diagnosed with Alzheimer’s disease be wise?
3. Anne is an 80-year-old woman. Throughout her life, other people have considered her to be wise. A few years ago, she was diagnosed with Alzheimer’s disease. In your opinion, is Anne still wise?

The Cronbach’s alpha for these three questions was .83. After answering these three questions, participants were asked to provide a rationale for their prior responses. They were asked to describe, in their own words, the relationship between memory loss, dementia, and wisdom. Thematic analysis was used to classify recurring patterns and themes within these responses (see Results section).

### Procedure

The measures described previously were included in a larger set of online study examining person perception. After providing implied consent, participants first completed an unrelated task in which they were shown a photograph and were asked to rate the likely competence of the pictured individual. Results from this task are reported in Barber, Lee, Becerra, and Tate (2019, Experiment 2) and will not be discussed further here. We next assessed participants’ aging attitudes using the Anxiety about Aging Scale (Lasher & Faulkender, 1993), and their knowledge about aging and AD using questions adapted from Berry, Williams, Thomas, and Blair (2015; see Appendix A in Supplementary Material). To ensure that participants were not choosing responses at random, within the Anxiety about Aging Scale we embedded the statement “I will show that I am reading the questions by choosing ‘*strongly agree*’ for this question” and eliminated responses from participants who did not select the appropriate response (see Participants section). Participants next provided demographic information about themselves and completed a second unrelated questionnaire about “borrowing” memories (i.e., telling another person’s story as if it was your own), which will not be discussed further here. Following this, we assessed implicit theories about wisdom using the measures described previously. Approval of the data collection using these procedures was obtained from the Institutional Review Board at San Francisco State University. Approval for the analyses was obtained from the Institutional Review Board at Georgia State University.

## Results

### Quantitative Responses

As given in Table 1, the quantitative data showed that participants overwhelming thought that it was possible to be wise despite age-related cognitive declines and despite a diagnosis of AD. More specifically, 78% of participants thought people who have experienced cognitive decline and who were prone to senior moments could be wise. Whereas only 65.8% thought someone with a diagnosis of AD could be wise, this rose to 75.8% when it was specified that the person in question was an 80-year-old woman who had previously been considered wise. Correlations among the responses were high and are given in Table 2.

**Table 1.** Proportion of Respondents Selecting Each of the Five Response Options for the Three Quantitative Questions About the Relationship Between Wisdom, Cognitive Decline, and Alzheimer’s Disease

*N* = 1,519	Definitely yes	Probably yes	Might or might not	Probably not	Definitely not
1. Can someone who has experienced cognitive decline and who is prone to “senior moments” be wise?	.395	.385	.177	.024	.003
2. Can someone who has been diagnosed with Alzheimer’s disease be wise?	.313	.345	.230	.080	.016
3. Anne is an 80-year-old woman. Throughout her life other people have considered her to be wise. A few years ago she was diagnosed with Alzheimer’s disease. In your opinion, is Anne still wise?	.363	.395	.168	.052	.005

**Table 2.** Participant Characteristics as a Function of Their Qualitative Responses

Participant Characteristic	Wisdom remains (*n* = 766)	Wisdom lost (*n* = 247)	Comparison
Age	46.40 (15.10)	51.08 (16.26)	*t*(1,011) = −4.16, *p* < .001
Education			χ(7) = 17.54, *p* = .014
Less than high school	1.6%	0.4%	
High school/GED	21.4%	13.8%	
Some college	22.0%	22.3%	
2-year college degree	11.6%	13.0%	
4-year college degree	28.8%	27.1%	
MA degree	11.6%	17.4%	
PhD degree	0.9%	2.0%	
JD or MD degree	2.1%	4.0%	
Gender			χ(1) = 18.58, *p* < .001
Male	22.7%	36.6%	
Female	77.3%	63.4%	
AAS—Fear old people	2.49 (1.07)	2.71 (1.08)	*t* (1,011) = −2.84, *p* = .005
AAS—Physical concerns	2.94 (1.15)	2.98 (1.14)	*t* (1,011) = −0.57, *p* = .571
AAS—Physical appearance	3.51 (1.29)	3.48 (1.25)	*t* (1,011) = 0.32, *p* = .749
AAS—Fear losses	4.27 (1.22)	4.41 (1.18)	*t* (1,011) = −1.51, *p* = .131
Knowledge aging	3.34 (0.95)	3.36 (0.89)	*t* (1,011) = −0.37, *p* = .711
Knowledge aging vs AD	3.01 (1.15)	3.13 (1.11)	*t* (1,011) = −1.39, *p* = .166
Known someone with AD?			χ(2) = 0.03, *p* = .985
No	24.0%	23.5%	
Not sure	10.1%	10.1%	
Yes	65.9%	66.4%	
Residential work with older adults			χ(1) = 1.55, *p* = .213
No	65.8%	70.0%	
Yes	34.2%	30.0%	
Frequency older adult interactions	3.51 (2.00)	3.27 (2.00)	*t* (1,011) = 1.63, *p* = .103
Valence older adult interactions^b^	5.76 (1.03)	5.58 (1.10)	*t* (395.30) = 2.27, *p* = .024

*Note.* AAS = Anxiety about Aging Scale (Lasher & Faulkender, 1993); AD = Alzheimer’s disease. We limited these analyses to individuals whose qualitative responses were either consistent with the notion that wisdom is preserved despite cognitive declines/AD or were consistent with the notion that wisdom is lost because of cognitive declines. Participants who gave responses that were coded as consistent with aspects of both of these themes, or whose answers were coded as consistent with one of our excluded themes (footnote b) were excluded from these analyses.

^a^We limited this analysis to only individuals who self-identified as male or female.

^b^Because the Levene’s test for equality of variance was significant (*p* = .017), equal variances were not assumed.

### Qualitative Responses

Participants were also provided an opportunity to justify their responses. On average, justifications were brief and an analysis using the Linguistic Inquiry and Word Count (LIWC2015) program (Pennebaker, Boyd, Jordan, & Blackburn, 2015) found the median response length was 21 words (*M* = 27.82, *SD* = 28.16, range: 0–488). The response length was not significantly related to participant age, *r* = .033, *p* = .193, but women wrote longer responses (*M* = 29.14) than did men (*M* = 24.41), *t* (1,512) = 2.92, *p* = .004, *d* = .18.

These qualitative responses were read by the research team to identify recurring patterns and themes, and a codebook was collaboratively developed (Supplementary Material). Using this codebook, all responses were categorized by the first author. A randomly selected subset of 334 responses was also coded by the second author to ensure consistency. Although we allowed for the possibility that a given participants’ responses would be consistent with more than one code, this was relatively uncommon and applied to only 55 participants (3.6%). Statistics indicated adequate consistency between the two raters (for responses receiving only one code: Cohen’s kappa = .74).

Of the 1,519 participants, 442 (28.6%) provided responses that did not fit our coding scheme. This included participants who stated that they did not know how wisdom, cognitive decline, and dementia related to one another; who stated that these concepts all describe older adults; who stated that these concepts all related to the brain and/or cognition; or who defined each concept rather than describing their relationship(s) to one another.

Of the remaining 1,077 responses, 791 of them (73.4%) were consistent with the notion that wisdom could be retained despite cognitive declines and 275 of them (25.5%) were consistent with the notion that wisdom would be lost. Within these two large categories, we also coded for the most common justifications, which are described subsequently.

Our coding scheme also included two additional broad categories, but these responses were rarer than expected: 13 responses (1.2%) were consistent with the idea that cognitive declines lead to a partial, but not complete, loss of wisdom and 22 responses (2.0%) were coded as stating that the relationship between cognitive declines and wisdom varies across individuals. These less-common response categories will not be discussed further.

#### Wisdom is preserved despite cognitive declines and AD

We observed three common justifications for the belief that wisdom could coexist with cognitive decline and AD: (i) Wisdom is not lost because it is evidenced in lucid moments, (ii) Wisdom is an immutable characteristic that is impossible to lose, and (iii) Wisdom is not lost because it has no relationship with cognitive capabilities. Below we describe each in more depth.

1. Wisdom is not lost because it is evidenced in lucid moments

Of the participants who thought wisdom could be maintained despite cognitive declines, 158 respondents justified this by describing how people with AD can have lucid moments in which you catch glimpses of their wisdom. For example, one participant wrote: “I am confident that wisdom is not lost. There may be periods (long or short) of lack of clarity, but I cannot count the number of times a memory or thought comes their minds that is truly wise and true.” Likewise, another participant wrote that “even if a person has dementia, they can still have lucid moments. Therefore, depending upon when a question is asked, wisdom may be expressed in their words.” As a whole, these responses described day-to-day fluctuations in the ability to act wisely, but took moments in which wisdom was manifested as evidence that it still existed. These moments did not have to frequently occur. For example, one additional participant wrote:

I had a grandmother that had Alzheimer’s and she was still wise when she wasn’t effected [*sic*] by the Alzheimer’s. I just had to catch her in the moments. This of course required a lot of patience.

2. Wisdom is an immutable characteristic that is impossible to lose

Whereas responses in the previous category indicated a belief that wisdom can be present even when wise actions are rare, other participants believed that wisdom could be present even if wise actions *never* occur. In this set of 261 responses, wisdom was often seen as a core trait of a person that is either an innate quality (e.g., one participant described this as “something you’re born with”) or is something that develops over time but becomes a core part of a person’s essence or soul (e.g., another participant stated that “wisdom becomes part of character”). As such, these participants viewed wisdom as immutable. Participants wrote that “wisdom once achieved can never be lost,” that “once wise always wise,” and that “wisdom is something someone inherently has and gains over time—once you have it, it cannot be taken away.”

Within this set of 261 responses, it was also common to distinguish between expressing wisdom and possessing wisdom. For example, one participant wrote that you do not lose wisdom when you get dementia, “you just lose the ability to express it.” Likewise, another wrote that “just because you can’t see on the surface her wisdom doesn’t mean it doesn’t dwell below or is instilled inside her soul for only her to experience inside herself.” Thus, among these participants, manifestations of wisdom were not a requirement for the possession of wisdom.

It was also common for these participants to distinguish between the availability and accessibility of wisdom. Some of these participants described people with dementia as still having wisdom available, but being less able to access it. For example, one participant wrote: “You have gained the wisdom throughout your life, cognitive disease may impaire [*sic*] your ability to access these traits, but doesn’t erase them.” This sentiment was present in other responses. Another participant stated:

I feel that the wisdom they had before the onset of these cognitive losses is still in their minds—it just may not be able to be “retrieved” by that person at the time they need to “retrieve” it. I guess I think of it as part of your soul or being—it’s always there regardless of your cognitive state.

Finally, within this category of responses, it was also common to distinguish between characteristics of disease processes and characteristics of the person affected by the disease. Participants were often emphatic that diseases, such as AD, cannot change a person’s true identity. Even when the actions of a person with AD are unwise, this should be attributed to characteristics of the disease, which is acting as a mask and hiding the true characteristics of the person. For example, one participant stated that “the disease just masks the person it affects” but “doesn’t fully change them.” This overall sentiment is reflected well in the following response:

Memory loss and or dementia do not define a person, these impairments are simply a diagnosis, nothing more. Who a person is does not change when they are diagnosed with dementia, rather, the simple fact is that they are the same person who will now need the help of others to make it through the remainder of their life. therefore, I do not see a person who has dementia as less wise than when they were healthy, rather, they are the same person with new needs.

3. Wisdom is not lost because it has no relationship with cognitive capabilities

Of participants who thought wisdom could be maintained despite cognitive declines, our final subcategory consisted of people who stated that wisdom and cognition are unrelated (e.g., one participant wrote that “being wise has nothing to do with memory”). This set of 348 participants often viewed wisdom as having wider parameters than cognitive capabilities, and as such, did not think that cognitive declines necessitated wisdom declines. These participants tended to focus on memory abilities, and stated that the “loss of memories does not take away a person’s capacity for truth and emotional intelligence,” that “just because you lose your memory doesn’t mean you lose your morals,” and that “wisdom has nothing to do with the mind, but everything to do with right relationship [*sic*], reverence, & obedience to what God says is true!”

Within this set of responses, there was also a belief that the forms of memory lost with age and/or dementia are different from the forms of memory required for wisdom. For instance, one participant wrote that with cognitive decline or AD “past memories tend to stay but new experiences tend not to stick. The wisdom from years of experience can still remain fresh.” Likewise, another wrote that “someone can experience memory loss and lose memories of moments of their life. However, they can still retain information that they will keep forever, such as knowing how to do something.” In general, these respondents suggested that impoverished memory for recent events is irrelevant for determining whether a person can possess wisdom. Rather, these participants viewed wisdom as instead being dependent upon memory of remote personal events and/or upon semantic memory processes. For example, one participant wrote:

While someone with dementia will experience memory loss, they may still have wisdom. If I may speak from a personal standpoint, my grandmother has dementia. I see her memory loss gets more extreme each day, but she’s still very wise. She may not remember something you told her 30 seconds ago, but she still remembers lessons in life in which she learned something about herself. She always tells me memories she has from her childhood and young adulthood. Even though I have already heard all of her memories, I still enjoy hearing them because they help remind her that even though she has dementia, she is still wise. I remind her everyday that she’s not “stupid,” she’s just forgetful. So while someone may have dementia and/or memory loss, that doesn’t diminish their wisdom.

#### Wisdom is lost due to cognitive declines

In contrast to the prior responses, there were also participants who believed cognitive declines lead to wisdom declines. These participants all described wisdom as requiring cognitive capabilities and/or memory of life experiences. However, some participants also noted that the relationship between wisdom and dementia depended upon the severity of cognitive decline. To capture this distinction, when participants noted that wisdom requires cognitive capabilities, we separated the responses based upon whether or not they also included a description of a graded process. These two codes were mutually exclusive.

1. Wisdom requires memory of life experiences and cognitive capabilities

Of participants who thought wisdom would be lost, a common justification was that wisdom requires memory of life experiences and cognitive capabilities. These participants wrote that “if a person cannot remember their experiences then they lose wisdom previously gained,” that “wisdom implies remembering in order to share outcomes of revelant [*sic*] experiences” and that “if you can’t remember the past thoroughly you lose your wisdom because that is where your wisdom is sourced.” Within this set of 92 responses, some participants also noted that the loss of both cognitive capabilities and wisdom was inevitable due to physiological brain changes. For instance, one participant wrote that with “cognitive impairment there is an alternation of the functioning of neurons and the death of these cells which makes the person greatly lose memory and with it the wisdom.”

2. There is a graded scale: As cognitive capabilities decrease so too does wisdom

Of participants who thought wisdom would be lost, it was common to describe a graded process. Although these 151 respondents were generally optimistic that wisdom could be retained given mild cognitive declines, the majority believed that wisdom would eventually be lost as cognitive declines became severe. For example, one participant wrote that “a person can show a degree of forgetfulness but still have wisdom,” but “in the later stages of dementia the world around them fades away too and wisdom is pretty much gone.” Likewise, another participant wrote:

Memory loss does not necessarily mean that you are not wise. It depends on the seriousness of the memory loss. Not remembering peoples [*sic*] names and losing the keys does not impeded [*sic*] wisdom. However, serious dementia can impede wisdom since knowing how to handle day to day situations limits your ability to make wise decisions.

#### Knowledge of AD as a factor in believing that wisdom is preserved?

In coding the responses, it was also clear that participants varied in the extent to which they understood the symptoms of AD and other dementias. However, this did not seem to consistently predict their responses. There were some participants who believed that wisdom could be retained despite AD, but their responses showed misunderstandings about the symptoms of AD. For example, one participant wrote that “people with dementia do tend to have some short term memory loss problems, but certainly not the kind of memory loss that would change a person’s outlook so much that they could not [*sic*] longer be considered ‘wise’.” In contrast, other participants clearly understood the extent of cognitive decline that could occur with AD and yet still believed wisdom would remain intact. For example, one participant wrote that people with dementia “may have forgotten who their children are but are still able to give you very wise advice!”

In coding the responses, we also made note of any time that participants described a personal experience as part of their justification. However, inclusion of personal experiences did not consistently predict beliefs about how wisdom, memory, and AD/dementia interrelate. Some participants described personal experiences which led them to believe that wisdom is retained. For example, one participant stated that:

Wisdom is deep inside of you. It is a core of understanding that comes out. I took care of my 97 year old grandmother for the last seven years of her life, the last 14 months in my home. She had dementia. There were times she didn’t even remember if she had eaten, but she would tell me things that were very wise. That came from something in her that no amount of dementia could take away.

Likewise, another participant wrote:

I don’t think that you entirely lose everything. You can still be wise even if you do have memory loss or dementia. I’ve had 2 strokes, I can still remember things that happened back in the 70’s. But I have a hard time remembering what I did last week. As far as my wisdom, I think I’m as smart now as ever!

In contrast, other participants described personal experiences which led them to believe that wisdom (and more generally, the person’s former self) is erased with cognitive declines. For example, one participant wrote:

Sadly, one can lose their cognitive functioning to the point that their ability to reason and illustrate that wisdom is gone. My mother is such an example. I remember her wisdom and miss that about her. I still respect who she is but she is not wise now.

Likewise, another participant wrote:

My relationship with dementia is very personal. My granny had the disease and I watched her decline. It was incredibly heartbreaking to see her loss her able [*sic*] to communicate and take care of herself. She become [*sic*] a shell of the wise and loving granny she once was.

In total, 72 participants spontaneously described a personal experience that led to their answer. Of the 63 whose answers corresponded to one of our coding categories above (there were nine respondents who described a personal experience with memory loss and dementia, but did not describe how this related to wisdom), the only response category that they were more likely to use was that wisdom likely remains because you catch glimpses of it during lucid moments, *F*(1, 1,075) = 8.15, *p* = .004.

We also tested whether anxiety about aging, knowledge about aging, knowledge about AD, experiences with AD, or other participant factors (age, education, gender) varied based upon whether participants’ qualitative responses indicated that wisdom and cognitive decline can coexist. As given in Table 2, these analyses suggest that responses indicating a preservation of wisdom with cognitive decline/dementia were more likely to come from participants who were female, who were younger, who had less education, or who were less fearful of older adults. There were no significant relationships between the qualitative response category with knowledge of aging or AD, experience with AD, or frequency interacting with older adults.

## Discussion and Implications

In a study by Jeste and colleagues (2010), researchers with expertise in wisdom rated its essential qualities and were also encouraged to provide qualitative comments. Within these responses, one wisdom expert noted that an open question was: “What are the ways (other than developing dementia) in which one can lose wisdom?” (Jeste et al., 2010, p. 676). Results from the current study suggest that the assumption embedded in this question—that dementia leads to a loss of wisdom—is at odds with how many people conceptualize this relationship. The vast majority of participants in this study stated that wisdom could be present even in people with significant age-related cognitive declines or with AD. Below, we discuss how the three most common justifications provided to support this view fit with existing wisdom data and theories. When mismatches occur, they suggest that researchers’ theoretical propositions are not perceived by people as relevant in real-life settings. Furthermore, these mismatches may even suggest that researchers’ explicit theories are describing a construct other than wisdom (Yang, 2001).

### Wisdom Is Not Lost Because It Is Evidenced in Lucid Moments

Of the participants who believed that wisdom can coexist with cognitive decline, a common justification was to note that even people with severe AD have the potential to exhibit wise behaviors during lucid moments. These responses suggest a belief that wisdom, at the trait level, remains stable even though there is increased variability in wise behaviors at the state level (with wise behaviors occurring less frequently and unwise behaviors occurring more frequently). This implicit theory is consistent with research documenting substantial interindividual variability in wisdom-related performance (e.g., Grossmann et al., 2016; Pasupathi, Staudinger, & Baltes, 2001; Staudinger & Baltes, 1996; Zachry et al., 2018). Furthermore, given that this interindividual variability is greater among adolescents than adults (Pasupathi et al., 2001), it may also be true that interindividual variability is higher in old age, particularly among people with significant levels of cognitive decline. This would be consistent with a large body of research showing U-shaped developmental functions (Pauls, Macha, & Petermann, 2013; Siegler, 2004), and future research is needed to assess for this possibility.

However, this implicit theory assumes that occasional wise behaviors during lucid moments should be taken as evidence that a person (as a whole) is wise. This is at odds with how some researchers have conceptualized the relationship between states and traits. Although states and traits are not identical (Jayawickreme, Meindl, Helzer, Furr, & Fleeson, 2014), according to Fleeson and Jayawickreme’s (2015) Whole Trait Theory, state-dependent intra-individual fluctuations can be used to determine trait levels. The trait level will be the average around which the state-dependent fluctuations are most densely distributed. However, this theoretical view is inconsistent with the implicit theory that wisdom (as a trait) is present in people with AD even when wisdom-related behaviors are rare and limited to lucid moments. This implicit theory instead uses peak behavioral performance, rather than the average behavioral performance across time, when evaluating for the presence or absence of wisdom as a trait. More research is needed to evaluate when people rely upon peak versus average performance when ascribing traits.

### Wisdom Is an Immutable Characteristic

A second theme that emerged in the qualitative responses was an implicit theory that wisdom can never be lost. Because these participants viewed wisdom as immutable, they did not believe that it would be affected by cognitive declines or dementia. This essentialism view is similar to how people view other social categories, including race, gender (e.g., Haslam, Rothschild, & Ernst, 2000), personality characteristics (Haslam, Bastian, & Bissett, 2004), and mental disorders (Haslam & Ernst, 2002). It is also consistent with prior research showing that people in North America often hold essentialist beliefs about wisdom, viewing it as a fixed and unchangeable trait rather than as a malleable skill (Grossmann, in press).

However, prior research has also shown that there is cultural variability in this belief, and wisdom is viewed as more malleable by people from collectivistic cultures (Grossmann et al., 2019, as cited in Grossmann & Kung, in press). Building on this, it is possible that there are also cultural differences in implicit theories about the relationship between wisdom, cognitive decline, and dementia. In collectivistic cultures, it may be more common to see declines in cognition as leading to declines in wisdom. One limitation to the current study is that participants were all U.S. residents, and we did not assess cultural variation in responses. Future research is needed to assess this issue.

### Wisdom Is Unrelated to Memory Capabilities

Finally, in this study, there were also participants who endorsed the implicit theory that wisdom and cognition are not related constructs. This is at odds with many theoretical frameworks, which view wisdom as having a central cognitive component (e.g., Ardelt, 2000, 2003) or define wisdom as an intellectual virtue (Baltes & Smith, 1990, 2008; Baltes & Staudinger, 2000; Grossmann, 2017). However, this has more similarities to traditional Eastern interpretations of wisdom. Here, wisdom is seen as more intuitive and more reliant on emotions, with less overt intellectualization (Csikszentmihalyi & Rathunde, 1990). In fact, Lao Tze who founded Taoist philosophy actually viewed intellectualization as an obstacle for achieving wisdom. Wisdom instead was seen as exemplified by the integrative and intuitive way that children approach the world (Takahashi & Bordia, 2000). Although we did not include this as a coding category, a handful of the responses were consistent with this view. Some of these participants thought that a diagnosis of AD would actually lead to increases in wisdom because cognitive declines simplify the way we interact with the world and potentially lead to a new understanding of reality. Others noted that cognitive declines may also force people to rely more on remote memories, which they theorized were better suited for conveying wisdom.

### Implications for Implicit Theories About Dementia

These findings are also relevant for understanding people’s beliefs about how dementia affects the “sense of self” (i.e., the feeling that one is a unique and distinct person). In the literature, there is debate over this relationship (Millett, 2011). Some have suggested that dementia leads to an inexorable dissolution of the self until nothing is left (e.g., Davis, 2004). However, in contrast to this, there is also a growing literature suggesting that cognitive declines and dementia do not necessarily lead to a loss of the self (Strikwerda-Brown, Grilli, Andrews-Hanna, & Irish, 2019; see also Strohminger & Nichols, 2014), and this continued sense of self may be a beneficial coping strategy that can enhance well-being and self-esteem in people living with AD (e.g., Beard, 2004; Clare, 2002; Romero & Wenz, 2001). In line with this, our results show that most people believe wisdom can be preserved with dementia. Future research is needed to determine whether this view is also associated with better coping and life quality for either people living with AD or for their family or caregivers.

### Limitations

Although there are many strengths to this study, there are also limitations that should be noted. One issue that often arises in studies of implicit theories is whether the results have external validity. In this study, people often conceptualized wisdom as being present in AD, but are these conceptualizations correct? Although prior research has suggested that implicit theories of wisdom can have external validity in cognitively healthy adults (Sternberg, 1985, Experiment 3), it is unclear whether the same would occur for adults with cognitive declines or AD. Although this limitation should be addressed in future studies, it is still important to understand implicit theories as they are the lens through which we evaluate both ourselves and others (Bluck & Glück, 2005).

An additional study limitation is that we did not provide participants with a definition of wisdom. Following past research (Glück & Bluck, 2011), we chose not to provide a definition in order to examine how people conceptualize wisdom and its relationships with cognitive decline and dementia. However, a limitation of this approach is that some participants interpreted the concept of wisdom differently than is commonly accepted by researchers. For example, even though we explicitly stated that wisdom and intelligence are separate virtues, it was relatively common for participants’ qualitative responses to use these terms interchangeably.

An additional limitation is that our participants’ responses were very brief and were typically only consistent with one coding category. However, there is likely more nuance in many participants’ understanding of these relationships. Future research is needed to explore this possibility with more in-depth qualitative interviews. In doing so, it would also be informative to more systematically explore how views about these relationships are shaped by experiences with AD and other dementias.

A final limitation that should be noted is that there are differences between laboratory ratings of wisdom and spontaneous ascriptions of wisdom (Bluck & Glück, 2005). Participants in this study tended to agree that people with AD have the potential to be wise. This may be because we asked them whether people with AD can be wise (i.e., is it a possibility?) rather than to rate the likelihood that they actually are wise. It is also possible that if our participants had interacted with someone who has AD, they may not have spontaneously described that person as wise. Thus, this study likely overestimates the extent to which wisdom is seen as a virtue among people with cognitive declines and AD in the real world.

## Summary

We examined how everyday people conceptualize wisdom and its relationship to age-related cognitive declines and dementia as a means of shedding light on the essential characteristics of wisdom. Although prior research has examined implicit theories about how wisdom can be developed, to the best of our knowledge this is the first study to examine implicit theories about whether wisdom can be lost. Whereas prior studies have suggested that cognitive capabilities are seen as important for developing wisdom, the current results suggest that people believe that wisdom—once obtained—is not easily lost.

## Funding

This work was supported by internal department funds provided to the first author.

## Conflict of Interest

None reported.

## Supplementary Material

igaa010_suppl_Supplementary-Material
