# Supplementary material for: Wisdom Once Gained Is Not Easily Lost: Implicit Theories About Wisdom and Age-Related Cognitive Declines
Source: Innov Aging. 2020 May 4;4(2):igaa010. doi: 10.1093/geroni/igaa010 (PMC7197947; doi:10.1093/geroni/igaa010)
Supplement: igaa010_suppl_Supplementary-Material [file igaa010_suppl_supplementary-material.docx]

**Appendix A**

***Experiences with Aging Questionnaire***

1) How much do you know about the behavior and health changes that occur as people get older? *Response options from nothing at all (1) to a great deal (5).*

2) How much do you know about how healthy aging differs from Alzheimer’s disease and other forms of dementia? *Response options from nothing at all (1) to a great deal (5).*

3) I know, or have known, someone with Alzheimer’s disease. *Response options of yes, not sure, or no.*

4) Have you ever worked or volunteered in a nursing home, retirement home, hospital, senior center, or assisted living facility with older adults? *Response options of yes or no.*

5) How often do you interact with older adults (i.e., individuals who are 60 years of age or older)? Please select the statement that best summarizes your interactions. *Response options of several times per day, once or twice per day, several times per week, once or twice per week, several times per month, once or twice per month, several times per year, and once or twice per year.*

6) How would you describe your interactions with older adults (i.e., individuals who are 60 years of age or older)? Please select the statement that best summarizes your experiences. *Response options from always unpleasant (1) to always pleasant (7).*

**Supplementary Materials A**

**Codebook for Qualitative Coding of Wisdom Data**

Participants were asked the following: “In your opinion, what is the relationship between memory loss, dementia, and wisdom?” Responses are coded into one (or more) of the following categories. See notes below for when multiple codes are, or are not, appropriate.

**1: No answer for relationship between wisdom and cognition**

*Definition:* This code should be used when the participant does not clearly state a relationship between wisdom and cognition.

This includes instances where the participant does not respond or writes that they do not know. It also includes instances where the participant defines the concepts (dementia, wisdom, memory loss) but does not say how they relate to each other. It also includes instances where the participant writes that they are all related to thinking, to the brain, or to age. Single word answers, such as ‘probably’ or ‘aging’ should also be coded here.

*Multiple codes:* This code should only be used if codes 2 to 9 (described below) are not appropriate. This code can be used in conjunction with 10 (personal experiences).

*Examples ^1^^[[1]](#footnote-1)^*

- I don’t know too much about this subject
- All involve the brain
- With age comes wisdom and dementia
- Memory loss is forgetting things, dementia is not understanding, and wisdom is life experiences
- Some people are never wise. Loss of memory can happen to anyone. Dementia is a progressive medical condition.

**2. Wisdom is present -- Lucid moments**

*Definition:* This code should be used when participants state that wisdom is still present BECAUSE you still occasionally see it.

This code should only be used when the overall sentiment of the response is that wisdom can remain despite cognitive declines. It should also only be used when the response justifies this by describing a manifestation of wisdom.

The general sentiment of this code is that even though cognition has declined, there are sometimes moments where the person outwardly displays wisdom. These are often described by participants as “lucid moments”, “glimpses”, or “moments” of wisdom and clarity. This code can be used even when participants do not specifically use the term “wisdom”, but instead describe how in lucid moments you see things like a persons’ “smarts”, “brilliance”, or ability to give “good advice”.

*Multiple codes:* This code requires a response that specifies wisdom is retained despite severe cognitive decline. However, it is theoretically possible for this code to be given in conjunction with code 8 (There is a graded scale where cognitive decline leads to wisdom decline). Respondents may indicate that wisdom is preserved with mild memory loss, more likely to be lost with dementia, but still occasionally manifest during lucid moments.

*Examples:*

- Someone can have memory loss and dementia but still have moments of wisdom -- intermittently.
- People with Alzheimer’s can have moments of memory loss but other moments they are lucid and can have wisdom.
- I think even someone with memory loss can give great wisdom to others. There are still moments and areas of mental clarity even when someone is losing their mind. Even my grandmother who has significant dementia can still offer incredible insights.
- Wisdom can remain in moments of clarity even with those that have memory loss or dementia.

**3. Wisdom is present – It is an immutable quality**

*Definition:* This code should be used when participants’ responses imply that it is difficult – or perhaps impossible -- to lose wisdom.

This code should only be used when BOTH of the following are true:

1. The overall sentiment of the response is that wisdom can remain despite cognitive declines.
2. There is justification that wisdom remains by explicitly describing wisdom as something that can never be lost, or by implying that despite cognitive declines the wisdom remains but is not as easily accessible.

The general sentiment of this code is that wisdom is a trait or part of your essence. These respondents may describe Alzheimer’s disease as a “mask” that hides the person, but the core essence of the person (including their wisdom) still remains. These responses may also describe a person with cognitive declines as having problems “accessing” their wisdom; this implies that wisdom itself is not lost despite cognitive declines.

*Multiple codes:* This code differs from the previous (2. Wisdom – Lucid Moments) in that it does not require wisdom to ever be manifested.

*Examples:*

- You can’t lose your wisdom. You just don’t know how to use it correctly if your memory is failing you.
- I believe that wisdom can be deeply ingrained in our DNA and soul. Even though someone experiences memory loss, inherently they are still wise from past experiences, even if those memories become lost or foggy.
- Losing your memory from Alzheimer’s/dementia doesn't necessarily remove the part of yourself that makes you wise, but it can make it more difficult to show that wisdom toward others.
- Person may still be wise but lack the ability to act in accordance with the wisdom.
- Wisdom is still in the person, but is sometimes hidden in them because of their illness.
- Wisdom never fades.

**4. Wisdom is present – Wisdom and cognitive decline are unrelated**

*Definition:* This code should be used for responses that state wisdom remains with cognitive decline because there is no relationship between wisdom and age-related cognitive declines / dementia.

To receive this code, the overall sentiment of the response must suggest that wisdom can remain despite cognitive declines. This can then be justified in one of the following ways:

1. Participants’ note that wisdom and cognition/memory are not the same. They may say there is no relationship between these factors or they may say that cognition/memory loss does not mean wisdom loss.
2. Participants note that cognitive declines occur, but describe wisdom as being a non-cognitive quality more akin to spirituality, morality, or decision making.
3. Participants state that the type of cognition that declines with age or dementia is different than the type of cognition needed for wisdom.

Note that many of these responses acknowledge cognitive losses. They often begin with statements such as “even though there is memory loss” or “despite having dementia” or “although memory declines”. However, after acknowledging these losses, the response goes on to imply (or explicitly state) that they are irrelevant for the retention of wisdom.

*Multiple codes:* The key distinction between this code and the previous (3. Wisdom – Immutable quality) is that these responses must focus specifically on cognitive loss as being irrelevant to wisdom loss. They do not necessarily state that wisdom cannot be lost, but rather focus on the idea that wisdom and age-related cognitive declines or dementia are different constructs.

*Examples:*

- Wisdom is developed from our responses to past experiences. It is not related to any present cognitive impairments.
- You can still be wise even though you forget things.
- Just because they can’t recall everything doesn’t mean they don’t have some wisdom left.
- Even if you are forgetful, you can still understand about good decision making.
- There is no relationship between memory loss, dementia and wisdom. One can be wise even with memory loss and dementia
- Wisdom is imparted spiritually...memory loss doesn't rob you of it.
- You can still be wise if you have memory loss since wisdom is associated with compassion, ethics.
- With most dementia people retain what happened to them when they were younger the longest. So if they lived a life considered to be wise that value will remain with them to an extent, even as their shorter term memory declines.
- You can lose your memory and connectivity but you can still make spot on judgements and display humor.

**4. Wisdom is present – “Other” (Codes 2, 3, and 4 do not apply)**

*Definition:* This code should be used for responses that clearly describe wisdom remaining with cognitive decline, but either provide no justification OR provide a justification that does not fit with the prior coding categories. These responses are typically very short in length with minimal justifications.

*Multiple codes:* This code should only be used if codes 2, 3, and 4 (described above) are not appropriate.

*Examples:*

- Wisdom stays.
- She has many memories that give wisdom and since having dementia that can add to wisdom.
- Alzheimer’s does not mean they are stupid or have lost wisdom.
- People with memory loss or dementia can still be wise

**5. Partial loss of wisdom**

*Definition:* This code should only be used if the answer clearly states that cognitive declines and dementia take away some, BUT NOT ALL, of a person’s wisdom.

*Multiple codes:* Note that this code category deals with the amount of wisdom that can possibly be lost. This is different from code 8 below, which describes how the amount of wisdom lost varies based upon the severity of the cognitive decline. Responses that describe a graded scale of wisdom loss based upon cognitive loss should be assigned code 8 (There is a graded scale) rather than code 5 (Partial Loss). However, it is theoretically possible for both of these codes to co-occur.

*Examples:*

- Dementia takes away a bit of wisdom.
- With cognitive decline, some wisdom you retain, others you lose.
- A person who is considered wise before memory loss/dementia may still have some wisdom in certain areas.
- I do not feel that these conditions remove all of the wisdom that a person has obtained throughout their life.

**6. Individual differences in loss of wisdom**

*Definition:* This code should only be used if the answer clearly states that the relationship between wisdom and cognitive decline/dementia varies across people. This may be attributed to luck or unknown factors (cognitive declines lead to wisdom declines for some people but not others). However, participants may also have guesses about factors that cause cognitive declines to lead to wisdom declines.

*Examples:*

- The relationship between memory loss, dementia, and wisdom in my opinion can differ in people
- Varies - different correlation for different folks
- It depends on whether she still is willing to listen, learn, and grow. if she is willing, she's still wise; if she's given up, then no.
- There are varying degrees of these illnesses and each person has to be evaluated on an individual basis. some people still have wisdom although they may show the effects of these problems.

**7a. Wisdom is lost because it requires memory /cognition**

*Definition:* This code should be used for responses that clearly describe cognitive decline as causing wisdom decline.

To receive this code, participants should describe wisdom as requiring intact cognitive capabilities. Responses may state that memory of past life experiences are essential for wisdom, or may describe cognitive capabilities as being a requirement for acting in a wise manner. However, all responses will have the common feature of describing a causal relationship where cognitive declines lead to wisdom declines.

*Multiple codes:* See note below for 7b.

*Examples:*

- Because people who experience dementia or Alzheimer’s lose their own memories, it is hard for them to be "wise" because they are not remembering the experiences and advice that made them who they are.
- Wisdom tends to be gained from something that has already been experienced. The person would have to remember those experiences in a cognitive way in order to be considered wise or have wisdom.
- When individuals suffer from memory loss and dementia, they aren't able to handle even the most basic tasks in their day. It's difficult to incorporate wisdom in your life when you don't remember how to tie your own shoes. You've forgotten everything that made you a wise person.
- I believe if you can't remember what you have learned or gone through I believe you can lose the wisdom you have gained through your life
- In cognitive decline the person is unable to "tap in" to the things they used to know and remember. If you can't recall the things you knew and experiences you had that led to your wisdom, you are no longer wise unfortunately.

**7b. There is a graded scale where cognitive decline leads to wisdom decline**

*Definition:* This code should be used for responses that clearly describe a graded relationship between cognitive decline and wisdom decline.

Note that people may vary in how they think these graded scales unfold. Some may state that you can be wise with memory loss but not with dementia. Others may state that wisdom is present in early stages of dementia but is absent in later stages. Others may state that wisdom is present if you have either age-related cognitive declines or dementia, but that you can’t have both.

*Multiple codes:* This code differs from the previous (7a. Wisdom is lost because it requires memory/cognition) in that it provides a more nuanced description of the relationship between these constructs. Responses in the previous code were broad statements that without cognition you cannot have wisdom. Responses in this code instead focus on how this process unfolds. They may describe a linear relationship such that cognition declines are leading to wisdom declines over time. However, they may also state that wisdom can remain with age-related memory loss, but will be lost if the cognitive declines are severe enough to be classified as dementia or Alzheimer’s disease.

NOTE THAT ALTHOUGH IT IS POSSIBLE TO GIVE MULTIPLE CODES TO RESPONSES, THIS IS NOT TRUE IN THIS CASE. Responses coded as 7b (There is a graded scale) should NOT also be coded as 7a (Wisdom requires cognition).

*Examples:*

- There are different stages. As they progress I think their wisdom slowly fades away
- When you have memory loss that is minor, you can still be wise but if you have dementia in its later stages, you would lose your wisdom in being able to remember experiences and would no longer be wise even if you were when you were young
- There are different degrees of memory loss and dementia. Wisdom is from years and years of clarity. I believe you can have some memory loss and still have wisdom. Dementia has different degrees and the farther you are along in the disease, the less wisdom that you can remember.
- Senior moments or forgetfulness wouldn't seem to impede wisdom as those person's experiences and gained knowledge over the span of their life would still be accessible at times. Dementia depending on its severity seems like it might impede that persons clarity in understanding situations and/or drawing from experiences and lessons learned.

**8. Wisdom is lost – “Other” (Codes 7a and 7b do not apply)**

*Definition:* This code should be used for responses that clearly describe wisdom as being absent in dementia or with cognitive decline, but either provide no justification OR provide a justification that does not fit with the prior coding categories. These responses will typically be very short in length with minimal justifications.

*Multiple codes:* This code should only be used if codes 7a and 7b (described above) are not appropriate. The key difference between this and Code 9 (Wisdom is lost because it requires memory /cognition) is that these responses do not link the loss of wisdom to the loss of cognitive capabilities.

*Examples:*

- Wisdom shouldn’t be there.
- I could see people thinking that people with dementia or memory loss as less wise.
- Dementia may include memory loss, but also includes loss of wisdom

**9. Personal Experience?**

*Definition:* This code should be used for any responses that include a personal experience or anecdote. This does not have to be about family, but does have to be an actual experience with a person rather than book knowledge or media portrayals. It can also be about the self (own awareness cognitive declines).

*Multiple codes:* This code can be used in combination with any of the prior codes.

*Examples:*

- My mother and an aunt had dementia, but were still able to impart wisdom in the stories they told. The stories might ramble on, yet their wisdom came out in the way they wove it in with the tale.
- My father had dementia and he was a changed person, acting very different from his true self but that was entirely different than when he just had memory loss. Memory loss didn't really affect the way he lived or his ability to carry on with relationships, but the dementia affected how he behaved, talked, walked, and his judgement was a serious health and safety issue.
- My father also had memory loss. Although he had moments where he forgot things or people, he was still a very wise man and could function fairly well
- I feel no matter the age if they know what is going on still some they can be wise. Memory loss and dementia does not mean you lose it all. I have a friend who goes to church and has memory loss but still at church it comes from his heart and he remembers things well.
- I chose my answer based on my father-in-law, who had Alzheimer's. He lost his ability to talk and did not seem to know where he was most of the time. At one time, he was wise about several aspects of life, but due to mental decline, I think he forgot about everything.
- My grandma got sick and she was still kind and had moments of wisdom.

1. ^1^ Examples are taken from the participant responses, but have been edited for spelling and grammar. [↑](#footnote-ref-1)
